# Screening of self-assembled monolayer for aflatoxin B1 detection using immune-capacitive sensor

**DOI:** 10.1016/j.btre.2015.10.001

**Published:** 2015-10-27

**Authors:** Alvaro V. Gutierrez R, Martin Hedström, Bo Mattiasson

**Affiliations:** a Department of Biotechnology, Lund University, Lund, Sweden; b IIFB, FCFB, Universidad Mayor de San Andres, La Paz, Bolivia; c CapSenze HB, Medicon Village, Lund, Sweden

**Keywords:** Capacitive, Sensor, Aflatoxin, Self-assembled monolayer, Capacitance

## Abstract

A capacitive biosensor was used for detection of aflatoxin B1. Two different methods for cleaning gold electrodes were evaluated using cyclic voltammetry in the presence of ferricyanide as redox couple. The methods involve use of a sequence of cleaning steps avoiding the use of Piranha solution and plasma cleaner. Anti-aflatoxin B1 was immobilized on self-assembled monolayers (SAM). The immune-capacitive biosensor is able to detect aflatoxin B1 concentrations in a linear range of 3.2 × 10^−12^ M to 3.2 × 10^−9^ M when thiourea was used to form the SAM; 3.2 × 10^−9^ M to 3.2 × 10^−7^ M when thioctic acid was used. When the gold surface was isolated with tyramine-electropolymerization linear ranges of 3.2 × 10^−13^ M to 3.2 × 10^−7^ M and 3.2 × 10^−9^ M to 3.2 × 10^−7^ M where obtained, respectively. The results obtained show the difference in linear range, limit of detection, and limit of quantification when different self-assembled monolayers are used for aflatoxin B1 detection.

## Graphical abstract

**Figure:**
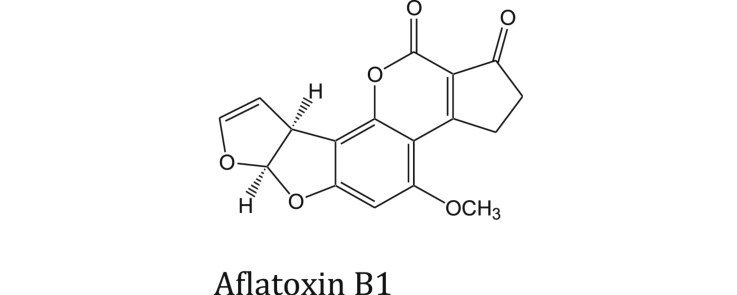

## 1 Introduction

Aflatoxins represent a major class of mycotoxins having deleterious impact in human and animal health. Aflatoxins are produced by *Aspergillus* fungi, section *flavi*, which includes *Aspergillus flavus* and *Aspergillus parasiticus* [1], [2], [3]. Studies of aflatoxins have shown mutagenic, teratogenic, and highly hepatotoxic and hepatocarcinogenic effects. Regulatory limits have been established in terms of concentration, which differs from country to country [4], [5]. The fungi are contaminating various crops and produce aflatoxins. Contamination of oil-rich crops such as corn, peanuts, cottonseed, and tree nuts [6] are common. Aflatoxin B1, which is a small hydrophobic molecule with a molecular weight of 312.3 Da is the major aflatoxin produced by toxigenic strains [7], [2].

The primary methods for aflatoxin detection are thin layer chromatography (TLC) [8], high performance liquid chromatography (HPLC) [9], and enzyme-linked immunosorbent assay (ELISA) [10]. TLC analysis is relatively economic, but is tedious and time consuming. HPLC analyses require extensive time for cleanup but similar as for TLC, the detection sensitivity is rather low. ELISA is the most commonly used method since the analytes can be detected relatively fast and quantified even at low concentrations among a multitude of other substances [10]. One new technique for detection of analytes at low concentration is the capacitive biosensor. This biosensor is highly sensitive, selective, requires low sample volumes and the samples do not need to be purified [11], [12], [13]. The principle of capacitive biosensor is based on measuring the change in capacitance caused by the change of dielectric properties when the target analyte binds to the immobilized biorecognition element (antibodies, receptors, etc.) attached to the sensor chip. The gold surface is isolated by a self-assembled monolayer and the biorecognition element is immobilized to that. When the analyte binds a resulting decrease of the capacitance is registered [14]. Capacitive biosensors have been used for the monitoring of a broad range of compounds/particles from heavy metal ions via glucose, to soluble proteins as well as larger aggregates such as virus particles and even microbial cells.

The capacitive biosensor is composed of three electrodes: a working electrode, a reference electrode and an auxiliary/counter electrode. The working electrode (i.e. transducer) is constructed as a gold surface onto which the sensing element, the ligand, is immobilized. Immobilization techniques based on different self-assembled monolayers (SAMs) [15], [16], [17], [18] have been adapted to the capacitive sensor surface in combination with various biorecognition elements [19]. The self-assembled monolayers of sulfur-containing compounds are used for insulation of the gold electrode. SAMs are formed from e.g. thioctic acid, thiourea or mercaptopropionic acid [17], An alternative technique is to use electropolymerization of e.g. tyramine [20] thereby creating an insulating layer and concomitantly introducing amino groups on the electrode surface. The amplitude of the signal registered from the sensor is a function of the surface area of the working electrode. Surface expansion might be achieved by adding gold nano-particles (AuNPs). Proteins are easily adsorbed to the nanoparticles, and that constitutes a convenient way of immobilizing antibodies. Such electrode surface has a good biocompatibility [21].

The effect of SAMs and electropolymerized tyramine as insulators of the electrodes was studied. Depending on the kind of insulator used when constructing the aflatoxin B1 sensitive electrode, the limit of detection and limit of quantification varied. Additionally, one simple cleaning method for flat gold surfaces was introduced and compared with the commonly used cleaning method for flat gold electrodes.

## 2 Materials and methods

### 2.1 Materials

**Fig. 1:**
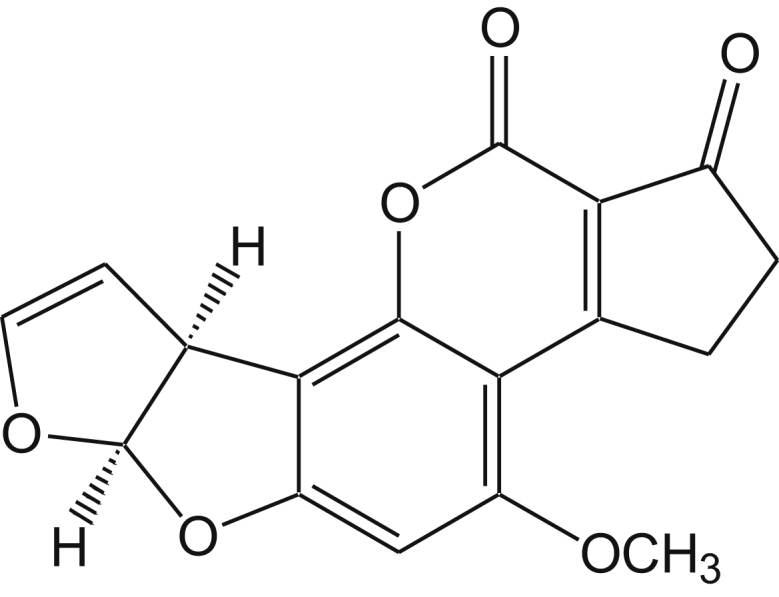
Aflatoxin B1 structure.

Anti-Aflatoxin B1, aflatoxin B1 (Fig. 1), thiourea, thioctic acid, tyramine, *N*-(3-dimethylaminopropyl)-*N*-ethylcarbodiimide hydrochloride (EDC) were obtained from Sigma–Aldrich (Steinheim,Germany), 1-dodecanethiol was obtained from Aldrich (Milwaukee, USA), . All other chemicals used were of analytical grade. All buffers were prepared from water treated with a MilliQ system from Millipore (Bedford MA, USA). This treated water is called MilliQ water in the rest of this paper. The buffers were filtered and degassed before use.

Samples from contaminated Brazilian nuts containing contaminated and non-contaminated nuts were kindly provided by Tahuamanu S.A. Company (Pando,Bolivia).

### 2.2 Methods

#### 2.2.1 Cleaning surface methods for flat gold electrodes

Disposable flat electrodes with a gold thickness 4000 Å and diameter of 3 mm were prepared as described by Teeparuksapun et al. [38] and cleaned using two different methods: (A) The gold electrodes were immersed first in acetone and then in ethanol under sonication for 1 min each, rinsed with water and dried with nitrogen gas before they were immersed in Piranha solution (sulphuric acid:hydrogen peroxide 3:1) under sonication for 1 min. Each step was followed by rinsing the electrode with MilliQ water (18 ΩM cm) and drying with pure nitrogen gas, finally the electrodes were placed into a plasma cleaner (Mod. PDC-3XG, Harrich, NY) for 15 min. (B) In the second method flat gold electrodes were immersed in acetone and ethanol under sonication for 5 min in each solvent, the two steps were followed by rinsing the electrode with MilliQ water (18 ΩM cm) and drying with pure nitrogen gas.

#### 2.2.2 Self-assembled monolayer and electropolymerization

**Fig. 2:**
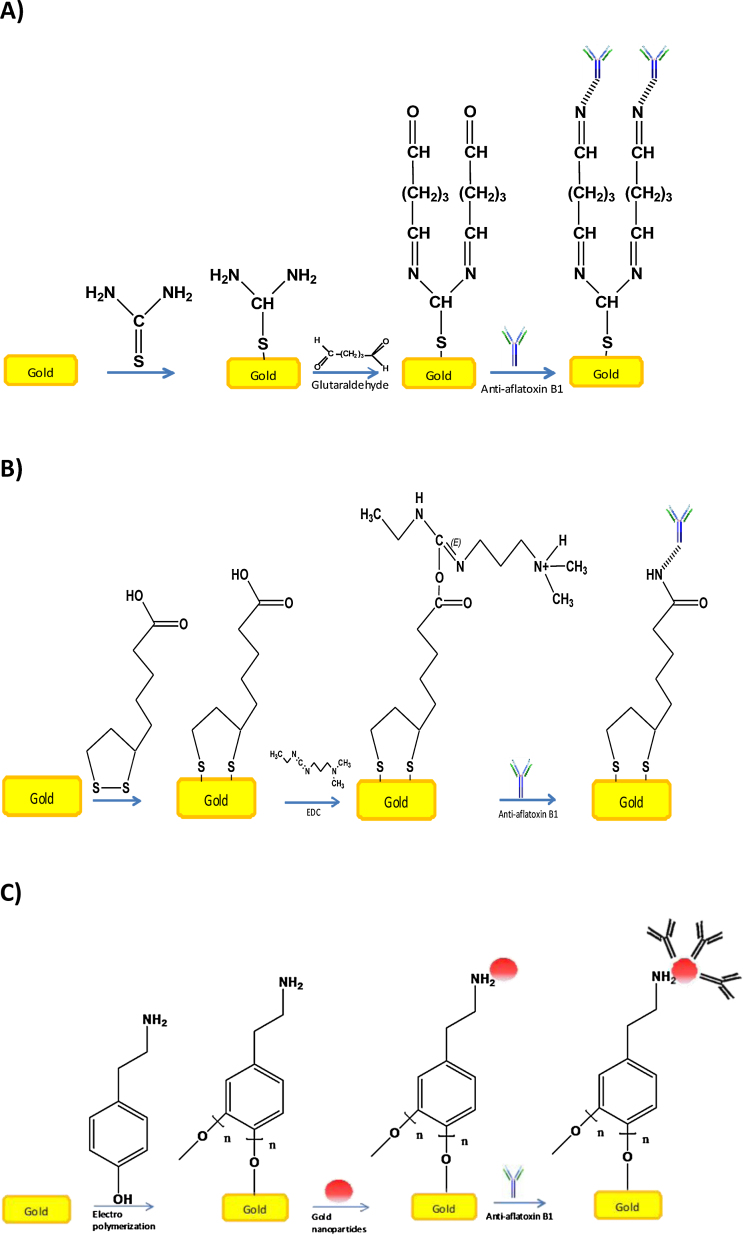
Insulation of flat gold electrodes and immobilization of antibodies on electrodes insulated by (A) thiourea, (B) thioctic acid, and (C) tyramine.

The cleaned flat gold electrodes recently rinsed with MilliQ water(18 ΩM cm) and dried with pure nitrogen gas were immediately immersed in thiol solutions (thiourea or thioctic acid 250 mM dissolved in ethanol, Fig. 2A and 2B respectively), at room temperature for 12–18 h, rinsed with MilliQ water (18 ΩM cm) and dried with pure nitrogen gas [17].

Tyramine electropolymerization (Fig. 2C) was performed using two different solutions separately. The first solution was NaOH (300 mM) dissolved in pure methanol, and the second solution was potassium phosphate buffer (10 mM):ethanol in a rate of 3:1. Tyramine (100 mM) was prepared in each solution and electropolymerized on the gold surface electrode. Electrodes were rinsed with MilliQ water (18 ΩM cm) and dried with pure nitrogen gas. The tyramine electropolymerization was carried out in a range of 0–1.5 V vs Ag/AgCl reference electrode for 15 scans using (8 PGSTAT12, Eco Chemie, The Netherlands), before the electrode was immersed in gold nanoparticles solution (Ø 2.6 nm) [22] for 18–24 h.

#### 2.2.3 Immobilization of anti-Aflatoxin B1

Anti-Aflatoxin B1, used as the biorecognition element, was immobilized covalently on SAMs built from thiourea and thioctic acid as follow: Flat gold thiourea electrode was immersed in a solution of glutaraldehyde 5% v/v in potassium phosphate buffer 10 mM for 20 min, rinsed with the same potassium phosphate buffer and dried with pure nitrogen gas. This chemical reaction is used for introduction of aldehyde groups that can be used to bind the anti-aflatoxin B1. A volume of 20 μL of anti-aflatoxin B1 (100 mg/mL) were added on the modified part of the flat gold electrode and incubated at 4 °C overnight. In the case of thioctic acid the carboxylic group was activated with 1% w/v of EDC in acetonitrile for 5 h, rinsed with MilliQ water (18 ΩM cm) and dried with pure nitrogen. A volume of 20 μL of anti-aflatoxin B1 in a concentration of 100 μg/mL was pipetted over the modified part of the flat gold electrode and incubated at 4 °C overnight. For the electrode modified with tyramine-gold nanoparticles, the electrode was rinsed with MilliQ water (18 ΩM cm) and dried with pure nitrogen, a volume of 20 μL of anti-aflatoxin B1 in a concentration of 100 μg/mL were pipetted over the reactive part of the gold electrode and incubated at 4 °C overnight. As a final step, the electrodes were treated with a solution of 1-dodecanethiol (10 mM) in ethanol for 20 min. This step is used to block pinholes in the insulation of the electrode surface.

#### 2.2.4 Capacitive measurement

The capacitive measurement was carried out using the newly developed technology [37] where current pulses are used instead of the potential pulses that most often are used. The advantage of using current pulses is that one can follow a linear change in potential as a result of the current pulse. In the older procedure, the potential pulse gave rise to a current signal that decayed logarithmically over time. The new concept give a far better stability of both baseline and of readings, and it contributed to an even higher sensitivity of the assay than what was achieved with the older procedure. The sensor chip is placed in a flow cell that is an integrated unit in a continuous flow system. The electrochemical flow-cell involves three electrodes, the working electrode that is the gold electrode which has been insulated and modified by immobilizing affinity binder structures on its surface [37]. The reference and an auxillary electrode are both made of platinum wire [12], [13], [37], [38].

The operating conditions for the immune-capacitive sensor were optimized by varying pH of both the regeneration buffer and running buffer. Regeneration buffer, glycine–HCl, was prepared in a concentration of 25 mM, adding HCl to reach pH 2.4, 2.2, and 2.0. In the case of running buffer 10 mM of potassium phosphate buffer at pH 7.0, 7.2, and 7.4 were prepared (Table 1).

**Fig. 3:**
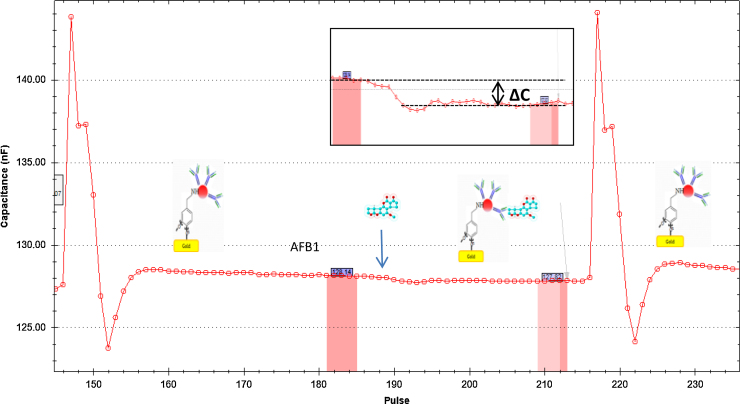
Capacitive sensing. The first peak shows the regeneration, then aflatoxin B1 is injected and binds to the antibody. Thereby producing a change in capacitance (Δ*C*), and finally one more regeneration to break the interaction Aflatoxin B1-antibody and make the electrode ready for a new assay cycle. Inserted is an amplification of the capacitance single as a result of injection of aflatoxin.

The flow rate of running buffer was set at 100 μL/min and the injection volume for regeneration buffer, samples, and standard was set at 250 μL. The total capacitance was calculated as follow:

$$\frac{1}{C_{tot}}=\frac{1}{C_{ins}}+\frac{1}{C_{rec}}+\frac{1}{C_{ag}}+\frac{1}{C_{DL}}$$

where *C*_tot_ is the total capacitance, *C*_ins_ is the capacitance due to the insulating layer (self-assembled monolayer, or the electropolymerized tyramine), *C*_rec_ is the capacitance caused by the recognition element (antibody), *C*_ag_ is the capacitance caused by binding the antigen (aflatoxin), and *C*_DL_ is the capacitance caused by the electrical double layer. As can be seen in the formula, the lowest capacitance dominates the total capacitance, for this reason the capacitance in the insulating layer must be as high as possible. Fig. 3 gives a schematic presentation of an assay cycle.

#### 2.2.5 Assessment of capacitive biosensor

In order to evaluate the analytical results obtained using the capacitive biosensor, the ELISA test kit was used as standard. ELISA test kit was purchased from Bioo Scientific Corporation (TX, USA), the procedure was followed as stated in the catalog provided by the supplier, and the readings were done using a microtiter plate reader (Biotek Elx808, Biotek instruments, USA) at a wave length of 450 nm.

#### 2.2.6 Sample treatment

Samples from contaminated and non-contaminated Brazilian nuts were ground, 5 g of ground nuts were mixed with 25 mL of methanol 70% v/v, shaken for 20 min and centrifuged at 4000 × *g* for 10 min. 1 mL of the supernatant was added to 1 mL of potassium phosphate buffer (10 mM).

#### 2.2.7 Selectivity

The commercial antibodies are well characterized, but still a test with another molecule (microcystine) was made in order to evaluate possible cross reactivity.

#### 2.2.8 Reproducibility

Aflatoxin B1 was detected repeatedly for 30 cycles. The reproducibility was evaluated by monitoring the change in capacitance using a standard target concentration of 10^−9^ g/mL for electrodes prepared for thioctic acid and tyramine (potassium phosphate/ethanol), and 10^−12^ g/mL for electrodes prepared with thiourea and tyramine (methanol/NaOH).

## 3 Results and discussion

### 3.1 Gold surface cleaning methods

**Fig. 4:**
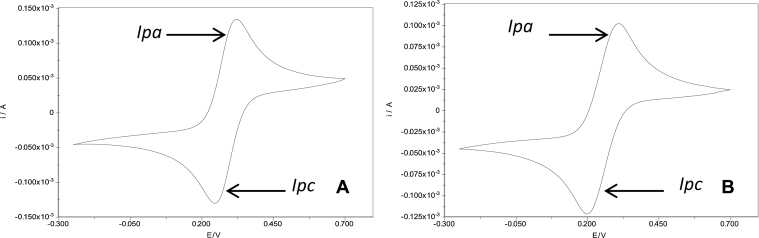
Cyclic voltammetry (CV) to evaluate the effect of the gold surface cleaning methods. (A) Method one. (B) Method two. *Ipa*: Anodic peak current. *Ipc*: Cathodic peak current.

Two different cleaning methods for flat gold electrodes were tested. The two methods involve almost the same reagents and procedure, but they differed with regard to use of Piranha solution (sulphuric acid:hydrogen peroxide 3:1), which is a toxic and harsh compound mixture, and plasma cleaner for method one, but not for method two, as mentioned in materials and methods. After cleaning, the electrodes were characterized using cyclic voltammetry with a potential range of −250 mV to 700 mV vs Ag/AgCl reference electrode for three cycles. Ferricyanide (K_3_[Fe(CN)_6_]) was used as permeable redox couple for both methods to evaluate the cleanliness of the surfaces [23], [24] (Fig. 4).

Cyclic voltammetry is a very versatile electrochemical technique, ideally suited for quick search of redox couples present in the system. When the working electrode potential is more positive than that of a redox couple present in the solution, the corresponding species may be oxidized (*Ipa*). Similarly, on the return scan, as the working electrode potential becomes more negative than the reduction potential of a redox couple, reduction may occur (*Ipc*). Fig. 4 shows the behavior of anodic and cathodic current which is common when clean electrode surface is present [25]. The differences between the two CV profiles were very small and therefore method two was used for all the experiment in this report.

### 3.2 Self-assembled monolayer and electropolymerization

Self-assembled monolayers on gold surfaces have been shown to be useful for insulation of the surface [17], [26] and for introduction of groups that can be used for subsequent immobilization of receptor structures. Self-assembled monolayers are also known for high capacitance values [27]. The generation of a functional surface of self-assembled monolayer is made to attach biological molecules (e.g. antibodies) for biosensor construction [17], [28]. In this study two different thiol-compounds, thiourea and thioctic acid, were used to obtain an insulating layer with suitable groups for immobilization of affinity reagents. The other approach of achieving both insulation and introduction of reactive groups is via electropolymerization. In this case tyramine was polymerized as a thick layer on the electrode surface. The free amino groups on the polymer were later used for immobilization of antibodies.

The efficiency of coverage of the surface is an important point when constructing a sensor chip. In a study on detection of resistance genes in multiresistent bacteria we demonstrated that electropolymerization gave a tighter coverage, and thus a better insulation. This is then seen in higher sensitivities in the analyses.

### 3.3 Optimizing pH of both regeneration buffer and running buffer

**Fig. 5:**
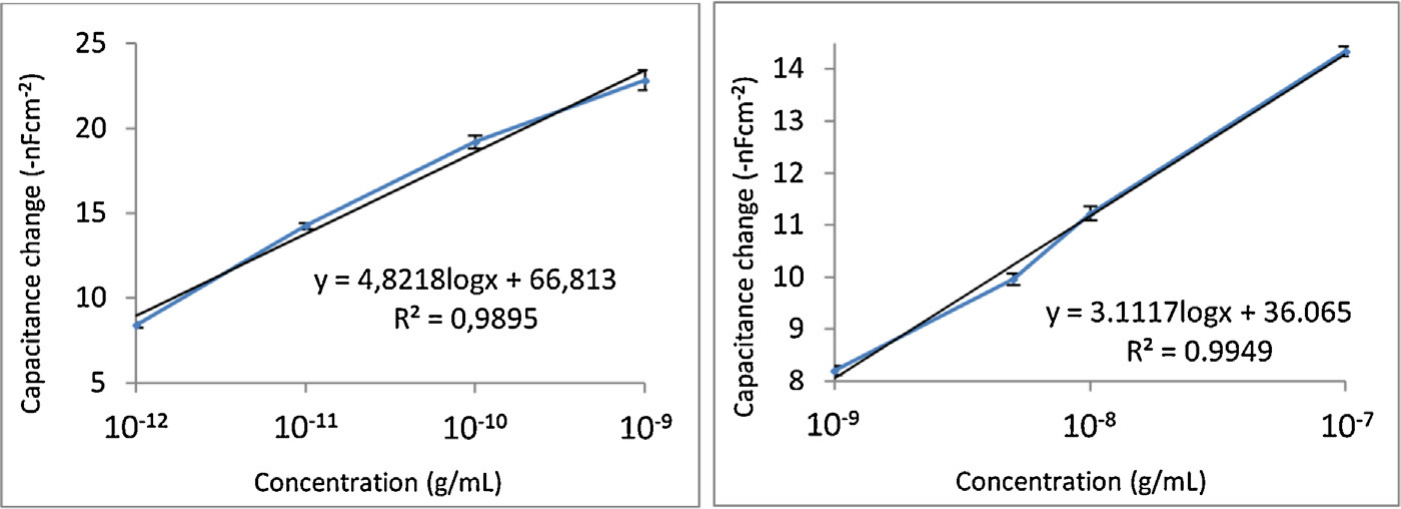
Calibration curves of aflatoxin B1 when assays with sensor chips insulated with SAMs of (1) thiourea and (2) thiotic acid as self-assembled monolayer with antibody immobilized to the insulating layer.

**Fig. 6:**
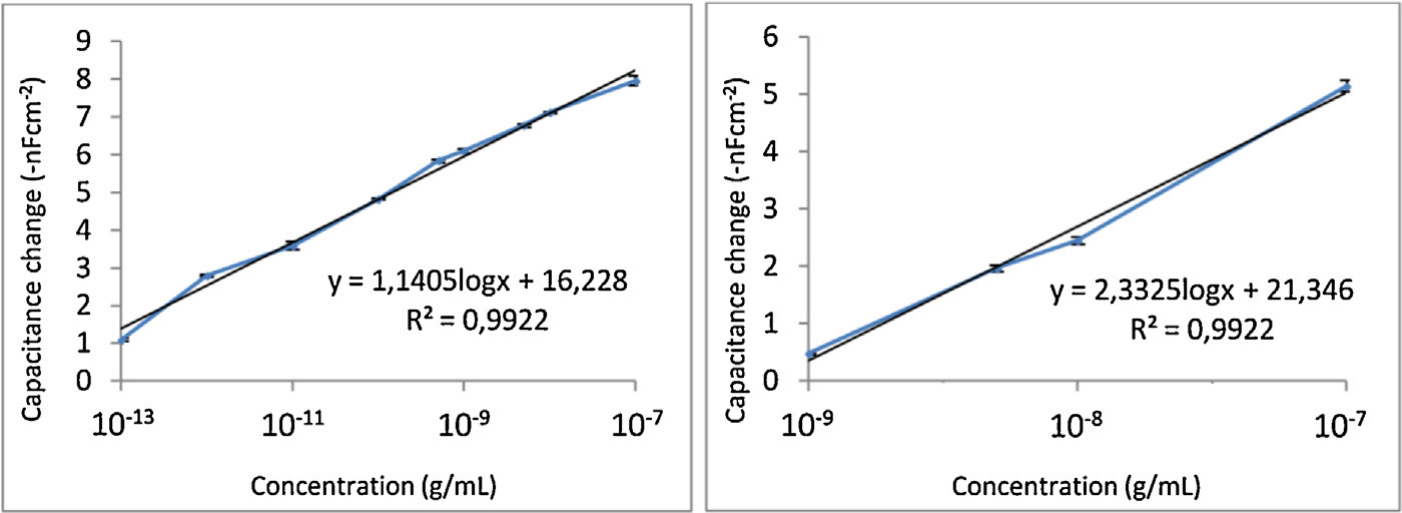
Calibration curves of aflatoxin B1 when assays with antibodies immobilized on polytyramine as the insulating layer. Two different types of solutions of tyramine were used for the electropolymerization: methanol/NaOH solution for tyramine electropolymerization (left), and ethanol/potassium phosphate buffer solution for tyramine electropolymerization (right).

Different concentrations of aflatoxin B1 were injected in the capacitive immune-sensor system in order to determine the linearity between response and concentration of the target and the limit of detection for aflatoxin B1 for sensor chips covered with the different insulators. Three injections for each concentration were made for all the calibration curves (Fig. 5, Fig. 6). However, to establish the ideal pH-values of running buffer and regeneration buffer, three running buffers with different pH and three regeneration buffers were tested as seen in Table 1.

The running buffer with a pH 7.4 was chosen and the regeneration buffer at pH 2.0. The regeneration buffer needs to break the molecular interactions between the antibody and the aflatoxin B1. These interactions between the antigen and the antibody involve hydrogen bonds and hydrophobic interactions [18]. The common regeneration buffer used for the release of other molecules than aflatoxin B1 has a pH 2.5 [12], [13], [26]. Crucial parameters when selecting the dissociation buffer are stability of the immobilized antibodies, effect of the dissociation buffer on the insulating layer and strength in binding between the antigen and the immobilized antibodies.

### 3.4 Calibration curves of capacitive biosensor for aflatoxin detection

Thiourea is considered as a molecule with low environmental impact, easy to handle and with characteristics to be strongly adsorbed on the gold surface. Previous studies when thiourea has been used have been focused on detection of different molecules [17], [29], but in most cases for high molecular weight molecules. Thioctic acid plays an important role in specificity, sensitivity, reproducibility and recycling ability in the performance of the immunosensor [30].

Analysis of aflatoxin B1 with thiourea forming the SAM has given analytical results with a linearity between concentration and response in a range of 10^−12^ g/mL to 10^−9^ g/mL (equivalent to 3.2 × 10^−12^ M to 3.2 × 10^−9^ M) for detection (fig. 5.) with a correlation coefficient of 0.9895 (*n* = 3). Thioctic acid has shown a linear range for calibration curve of 10^−14^ g/mL to 10^−9^ g/mL (equivalent to 3.2 × 10^−14^ M to 3.2 × 10^−9^ M) for aflatoxin B1 detection (Fig. 3) with a correlation coefficient of 0.9949 (*n* = 3) (Fig. 5).

Tyramine was electropolymerized on the electrode surface and the polymer film produced is considered to be strongly adhering to the electrode surface. It is the phenol moiety participating in electropolymerization, since the amino group is separated with two methylene groups from the phenol ring [20]. Electropolymerization was carried out in a potential range of 0–1500 mV vs Ag/AgCl reference electrode for both methods. The first method uses methanol/NaOH solution, which has shown to dissolve the tyramine faster than potassium phosphate buffer/ethanol solution. The first method gave an electrode surface which after modification was used in assays. It gave a linear range of 10^−13^ g/mL to 10^−7^ g/mL (equivalent to 3.2 × 10^−13^ M to 3.2 × 10^−7^ M) for aflatoxin B1 detection (Fig. 6, left). The second method gave a linear range of 10^−9^ g/mL to 10^−7^ g/mL (equivalent to 3.2 × 10^−9^ M to 3.2 × 10^−7^ M), as can be seen in Fig. 6 (right). In both cases with a correlation coefficient of 0.992 (*n* = 3).

In order to control the measurement potassium phosphate buffer and microcystin, separately, were injected as samples, in both cases no capacitance response was noticed. However, when aflatoxin was injected clear changes in capacitance were observed. Even with small molecules, it is possible to detect binding as a change in capacitance. An interpretation of this fact is conformational changes taking place upon binding in the binding molecule, or the counter ions surrounding the antibody, or a combination of these [39], [40].

The capacitive immune-biosensor has successfully been used for the detection and quantification of aflatoxin B1, reaching high sensitivity. The most common detection schemes involve the use of antibodies [26], [28] as the immune-capacitive sensor. Self-assembled monolayer is a common method of electrode insulation and works in a proper way as linker between the electrode and the biorecognition molecule [17], [26]. Different self-assembled monolayer molecules were tested, thiourea, thioctic acid together with an electropolymerized layer of tyramine. Depending on the characteristics of self-assembled monolayers and electropolymerization solution, the linear range, limit of detection and limit of quantification can be different (Table 2). The main difference in tyramine electropolymerization is due the use of an alkaline solution (methanol/NaOH), which has the characteristic of self-limited growth in electropolymerization [20], [31]. Introduction of gold nanoparticles assists in expanding the available surface on the electrode, thereby making it possible to immobilize more antibodies. This leads to an increased sensitivity [13].

The limit of detection and limit of quantification were compared with data from other articles for the detection of aflatoxins as can be seen in Table 3.

### 3.5 Detection of aflatoxin B1 using ELISA kit

Standards solutions of aflatoxin B1 were prepared and measured using an ELISA test kit as well as the capacitive biosensor to evaluate how well the results from the two methods correlated. The calibration curve for ELISA test kit was performed measuring 5 different standard concentrations and the correlated concentrations are compared (Table 4).

### 3.6 Samples and similar molecules

Samples from the Brazilian nuts were also measured and compared between the two methods as can be seen in Table 4.

These concentrations were calculated taking into account the dilution factor for the ELISA test kit. However, one needs to keep in mind that the sample from Brazilian nuts might contain all the aflatoxins (G1, G2, B1, B2), whereas aflatoxin B1 represents 80% of the total aflatoxin concentration [2], [36], which might be the reason for the difference in concentration.

Cross-reactivity against the different aflatoxins is clear. The commercial antibody preparations were binding several of the aflatoxin variants, and therefore such a step does not give any useful information. Instead a model structure, microcyctin, with some similar functional groups was used to evaluate cross-reactivity (Table 5).

### 3.7 Reproducibility

Reproducibility of the sensor chips involving the antibody against aflatoxin B1 was evaluated for thioctic acid, thiourea, tyramine (potassium phosphate) and tyramine (methanol/NaOH).

**Fig. 7:**
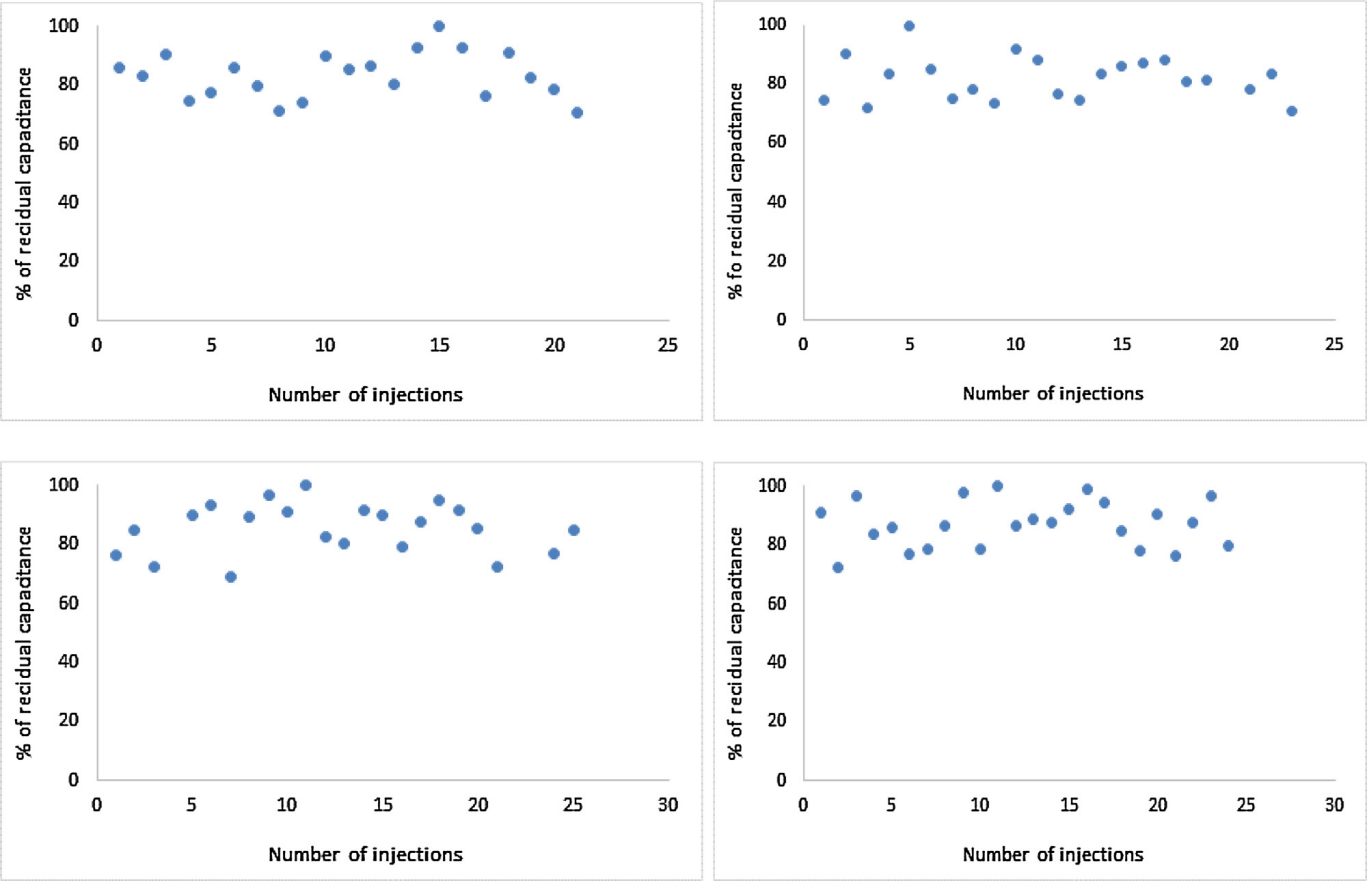
Reproducibility of the sensor chip using the antibody against aflatoxin B1. (1) Thiourea (up left) is reproducible for 21 cycles with a RSD of 9.3%. (2) thioctic acid (up right) is reproducible for 23 cycles with a RSD of 9.7% (3) tyramine (methanol/NaOH) (down left) is reproducible for 25 cycles with a RSD of 9.8%. (4) tyramine (potassium phosphate/ethanol) (down right) is reproducible for 24 cycles with a RSD of 9.12%.

As the recognition molecule was the same for all the experiments (antibody against aflatoxin B1) it was expected that the sensor chips would show a similar reproducibility (Fig. 7). The number of cycles (regeneration-injection-regeneration) goes from 21 to 25 with a RSD of <10% in all cases as the reduction in activity may be an effect of antibody denaturation on the surface or the loss of insulating layer [12].

## 4 Conclusion

Different methods to clean the flat gold electrodes for capacitive biosensor have been evaluated. It was clearly demonstrated that one can avoid using harsh chemicals such as Piranha solution and still keep a good analytical performance. However, the main result is that marked differences in analytical performance were observed between electrodes with self-assembled monolayers and tyramine electropolymerization over the gold surface electrode. The evaluation was made when analyzing aflatoxin B1. The results were compared with those from other type of biosensors for aflatoxin B1 detection, and showed lower limit of detection and limit of quantification for the capacitive biosensor. The best insulator corresponds to tyramine dissolved in methanol/NaOH with a linear range from 10^−7^ g/mL to 10^−13^ g/mL, presenting the currently best achieved limit of detection and limit of quantification (7.75 × 10^−15^ g/mL and 1.35 × 10^−14^ g/mL, respectively) for aflatoxin B1.

## Conflict of interest

The authors have declared no conflicts of interest.

## Figures and Tables

**Table 1:** Factorial design for selection of regeneration buffer and running buffer pH. Regeneration buffer: Glycine–HCl buffer 25 mM. Running buffer: phosphate buffer 10 mM.

Running buffer	7.0	7.2	7.4
Regeneration buffer			
2.0	X	X	X
2.2	X	X	X
2.4	X	X	X

**Table 2:** Limit of detection and limit of quantification for self-assembled monolayers and tyramine electropolymerization.

LOD	1.78 × 10^−14^	2.74 × 10^−12^	7.75 × 10^−15^	7.50 × 10^−10^
LOQ	2.93 × 10^−14^	4.31 × 10^−12^	1.35 × 10^−14^	8.49 × 10^−10^
	Thiourea (g/mL)	Thioctic acid (g/mL)	Tyramine (methanol/NaOH) (g/mL)	Tyramine (phosphate buffer/ethanol) (g/mL)


**Table 3:** Comparison of limit of detection (LOD), limit of quantification (LOQ) and linearity with other biosensing systems (*n* = 3).

	LOD	LOQ	Linearity (ng/mL)	Reference
SPR using antibodies	–	3ng/mL	3–98	[32]
SPR using antibodies	0.2 ng/g	–	1–10	[33]
SPR using single-chain antibodies fragment	0.37 ng/mL single scFv	–	0.37–12	[34]
0.19 ng/mL doble scFv	0.19–24
Enzyme immune biosensor	0.1 ng/mL	–	0.5–10	[24]
NRL array biosensor	0.6–1.4 ng/mL for nut products	–	–	[35]
SPR using neutrophil porcine elastase	0.97 ng/mL	3.1 ng/mL	1.67–17.8	[23]
Capacitive biosensor	7.75 × 10^−6^ ng/mL	1.3 × 10^−5^ ng/mL	10^−4^–10	This report

This capacitive biosensor for aflatoxin B1 detection has shown more sensitivity in most of the cases.

**Table 4:** Comparison of results from ELISA test kit and capacitive biosensor measurement. Values given are averages of 3 measurements (*n* = 3).

Aflatoxin B1 concentration (ng/mL)	Capacitive sensor (ng/mL)	ELISA (ng/mL)
0.1	0.11	0.096
0.5	0.51	0.52
1	1.03	0.98
5	4.93	Out of range
10	10.1	Out of range

**Table 5:** Sample measurement from Brazilian nuts, and the use of microcystin for possible crosslink reaction.

Sample	Biosensor measurement (ng/mL)	ELISA (ng/mL)
Brazilian nuts	43	48
Microcystin	No response	No response
